# What Is Moderate Drinking? Defining “Drinks” and Drinking Levels

**Published:** 1999

**Authors:** Mary C. Dufour

**Affiliations:** Mary C. Dufour, M.D., M.P.H., is deputy director of the National Institute on Alcohol Abuse and Alcoholism, Bethesda, Maryland

**Keywords:** moderate AOD use, standard drink, alcohol proof, wine, beer, distilled alcoholic beverage, survey, questionnaire, AOD use frequency, amount of AOD use, AOD abstinence, identification and screening for AOD use, self report, validity (research methods), reliability (research methods), AOD associated consequences, AOD impairment, risk assessment, literature review

## Abstract

Although the benefits and risks associated with moderate drinking have gained increasing attention in recent years from both researchers and the general public, no universal definition of moderate drinking exists. Most currently used definitions are based on a certain number of drinks consumed in a specific time period. Defining a “drink,” however, also is difficult because alcoholic beverages can differ substantially in their alcohol content, even within the same beverage category (e.g., beer, wine, or distilled spirits). Because international differences in drink definitions also exist, comparing studies from different countries is difficult. The development of a universal definition of moderate drinking is hampered further by variations in the way alcohol consumption levels and drinking patterns are being assessed (i.e., the survey methods and assessment modes used). Despite these problems, definitions of moderate drinking and drinking guidelines have been developed in the United States and other countries.

As documented by ancient texts, people have long been aware of both the harmful and beneficial effects of drinking alcohol. Research into alcohol’s effects, however, is relatively new, as evidenced by the fact that the National Institute on Alcohol Abuse and Alcoholism (NIAAA) was not created until 1971. Initially, alcohol researchers focused primarily on understanding alcoholism and on identifying effective prevention and treatment strategies. In recent years, however, moderate drinking also has become a topic of great interest and lively debate as researchers and the media have reported on the health benefits of moderate alcohol consumption. For example, studies have indicated that moderate drinking may be associated with reduced risk of heart attack, atherosclerosis, and certain types of strokes as well as a reduced risk of brittle bones (i.e., osteoporosis) in postmenopausal women.

To discuss adequately the potential benefits and risks associated with moderate drinking, one must first answer the question, What is moderate drinking? The meaning of the term “moderate” is highly subjective, however, and what one person considers to be moderate drinking, another person may view as heavy drinking. This variability makes it difficult to compare or interpret study findings regarding the consequences of moderate drinking. Accordingly, a definition of the terminology “moderate drinking” is needed to allow an informed discussion of the risks and benefits associated with such a drinking pattern.

Many current definitions of moderate drinking are based on a specific number of drinks consumed during a designated time period (e.g., per day or per week). This definition, however, raises the obvious question, What is a “drink” ? Another important question is, Why does it matter how a drink is defined? This article first reviews considerations relevant to defining a drink. It then describes several approaches to determining people’s drinking levels and patterns. Finally, based on that information, the article presents definitions of moderate drinking that are currently used in the United States and in other countries.

## What IS a Drink?

Both the definition and standardization of the term “drink” are relevant primarily in two settings: (1) commercial establishments that serve alcohol (e.g., restaurants and bars) and (2) alcohol research. The standardization of drink sizes has been a long-standing practice in alcohol-serving establishments. Commercial measures of alcoholic beverages, however, are heavily influenced by local drinking customs and regulations. In some countries, the serving sizes for various alcoholic beverages are mandated by law and, consequently, are uniform from one establishment to another.

In the United States, however, each bar, restaurant, or other establishment that serves alcoholic beverages can set its own standards, although establishments generally are consistent in the sizes of the drinks they serve. In private homes, drink sizes may vary even further. For beer, wine coolers, and similar alcoholic beverages, the serving size is most likely to be consistent across different households because a “serving” or drink often corresponds to one (standard size) can or bottle. For wine and distilled spirits (e.g., vodka and whiskey), however, the size of one drink is entirely up to the person pouring it and may vary from occasion to occasion.

Surprisingly, even in alcohol research no universally accepted standard-drink definition exists, although such a definition would be helpful for comparing the results of different studies. The lack of a definition is, to some extent, historically based. When alcohol-use surveys of the general population were first instituted, they focused primarily on the distinction between drinkers and nondrinkers (Clark and Midanik 1982). For example, in 1939 the Gallup surveys, which probably were the first surveys to measure drinking on a national level in the United States, included the following question regarding alcohol consumption: “ Do you have the occasion to use alcoholic beverages such as liquor, wine, or beer, or are you a total abstainer?” (Clark and Midanik 1982).

As alcohol survey research progressed and investigators became interested in assessing the consequences of various levels and patterns of alcohol consumption, scientists had to develop methods to quantify consumption more accurately. Over the 50 years that alcohol researchers in the United States and abroad have conducted surveys of alcohol consumption and alcohol-related problems in representative population samples, investigators have made great progress in survey research methodology, including the quantification of drinking levels. Nevertheless, no consensus currently exists as to the best methods or questions for eliciting reliable information on how much alcohol respondents drink. (Various currently used approaches for assessing drinking levels and patterns, as well as their advantages and disadvantages, are discussed in the section “Assessing Alcohol Consumption.” )

The definition of a standard drink is further complicated by the fact that in most studies of alcohol consumption, researchers are interested primarily in the effects of the alcohol contained in alcoholic beverages and not so much in the individual effects of various beverages. However, alcoholic beverages differ substantially in their alcohol content. Accordingly, a drink should be defined in terms of alcohol content, so that a drink of beer contains approximately the same amount of alcohol as a drink of wine or spirits. At first glance, this requirement appears to be a simple mathematical problem of comparing the alcohol contents of several beverages. In fact, however, such comparisons are rather complicated, because even within one beverage category (e.g., beer, wine, or distilled spirits), the alcohol contents may differ considerably.

In the United States, both the Federal tax code and the tax codes of individual States specify which beverages are classified as “ beer,” “ wine,” or “distilled spirits” and their alcohol content. According to those definitions, “ beer” includes strong beer (i.e., beer with an alcohol content greater than 3.2 percent), beer with an alcohol content of up to 3.2 percent, ale, malt liquor, and similar types of beverages. Similarly, “ wine” encompasses wine, vermouth, champagne, sparkling wine, cider, and related beverages. The broadest category is that of “distilled spirits,” which includes numerous beverages, such as gin, rum, vodka, whiskey, scotch, bourbon, and premixed cocktails.

The ranges of alcohol content for beer, wine, and distilled spirits vary somewhat from State to State. Significant variation also exists in the alcohol content of beverages within each of these categories. The typical alcohol content of beer is roughly 4.5 percent (by volume), but the alcohol content of light beers may be less than 3 percent, and certain craft-brewed beers or malt liquors may have an alcohol content of up to 9 percent or higher.

Similarly, the wine category encompasses fermented beverages with alcohol contents typically in the range of 11 to 14 percent. However, light wines may have an alcohol content in the neighborhood of 7 percent, whereas fortified wines (which include added distilled spirits) may range up to 24 percent alcohol by volume or higher. Also, wine coolers and hard ciders, which often are grouped with wines for tax and statistical purposes, typically have alcohol contents in the range of 5 to 7 percent alcohol by volume.

Finally, distilled spirits exhibit a wide range in terms of alcohol concentration. Typically, many familiar forms of distilled spirits (e.g., vodka, whiskey, gin, or rum) have alcohol contents of 40 to 50 percent (often expressed as 80 to 100 proof). Considerable variation may occur even within these categories, with the alcohol content of some varieties being as low as 30 percent and others as high as 75 percent. Liqueurs and cordials, usually grouped with distilled spirits, often are less concentrated than standard liquors. Grain alcohol, which is virtually pure ethanol, is often bottled at a concentration of 94 percent alcohol by volume.

In recent years, the alcoholic beverage market has become even more diversified. For example, beverages such as “ light” beer, “ light” wines, and wine or spirit coolers, which have slightly lower alcohol contents than the corresponding regular beverages, have been introduced. Conversely, both the relatively new “ ice” beers and “dry” beers have higher alcohol contents than do either regular or “ light” beers (Williams et al. 1997). Other beverages with higher alcohol content than the corresponding “regular” beverages, such as premium brand liquors, fortified wines, malt liquors, and locally produced beers and ales (i.e., microbrews), also have become more popular. Finally, large 40 ounce (oz) beer bottles have been introduced. Thus, a person drinking such a bottle may still report having had just one drink, although the amount consumed is approximately equivalent to the beer in three regular 12 oz bottles. These examples illustrate the difficulties encountered in determining and comparing actual alcohol consumption and the contents of various types of beverages for establishing a standard definition of a drink.

The alcohol contents of beer, wine, and spirits vary substantially within each category.^1^ To calculate and compare the alcohol contents of various beverages, however, scientists must select one conversion factor (or average alcohol content) for each category to reflect the alcohol contents of beer, wine, and spirits. One set of conversion factors that frequently is used in the United States defines average alcohol contents as follows (Doernberg and Stinson 1985):

- Beer—4.5 percent alcohol
- Wine—12.9 percent alcohol
- Spirits—41.1 percent alcohol.

The variability in the definition of a standard drink arises not only from differences among studies in the type of alcohol and the conversion factors used but also from the way in which the results are reported. For example, researchers can represent alcohol consumption as grams (g), milliliters (mL), or fluid ounces (fl oz) (American or British) of alcohol; beverage equivalents (e.g., “ beer equivalents” or “whiskey equivalents” ); or number of drinks, which can be variously defined. These different reporting methods can confuse the readers of various studies and complicate the comparison of study results. For example, people who are familiar only with the U.S. system of weights and measures will not know how much alcohol is present in a drink that contains 12 g alcohol. (For a conversion of milliliters of alcohol into grams and fluid ounces, see the table below.)

With all the confounding influences, not surprisingly, the sizes of standard drinks vary substantially among different countries. For example, a standard drink in Great Britain (i.e., a “unit” ) is equivalent to 8 g alcohol, whereas a standard drink in Japan (i.e., a “go” ) is equivalent to 19.75 g alcohol (Turner 1990). In the United States, the U.S. Department of Health and Human Services (DHHS) and the U.S. Department of Agriculture (USDA) have developed a commonly used definition of a standard drink that has been published in *Nutrition and Your Health: Dietary Guidelines for Americans* (DHHS and USDA 1995). According to that definition, a standard drink contains approximately 0.5 fl oz (or approximately 12 g) alcohol and corresponds to the following beverage amounts:

- 12 fl oz regular beer
- 5 fl oz wine
- 1.5 fl oz 80-proof distilled spirits.

**Table:** Conversion of Grams of Alcohol Into Milliliters and Into British and American Fluid Ounces

Country	Conversion^1^
Great Britain	1 fluid ounce = 28.41 mL
	1 fluid ounce = 28.41 mL × 0.785 g/mL = 22.30 g alcohol
United States	1 fluid ounce = 29.58 mL
	1 fluid ounce = 29.58 mL × 0.785 g/mL = 23.22 g alcohol

1 milliliter (mL) alcohol = 0.785 gram (g) alcohol.

SOURCE: Turner 1990.

In the scientific literature, the wide range of assumptions about what a standard drink is can produce highly divergent estimates of total alcohol consumption among respondents who report consuming the same number of drinks. In a review of 125 international epidemiologic studies that related various health consequences to different levels of alcohol consumption, Turner (1990) presented a striking example of the impact of methodological differences in converting the number of drinks of various alcoholic beverages to grams of alcohol. Turner based the comparison on fictional respondents who reported drinking one standard drink (as defined in each study) each of beer, wine, and spirits for a total alcohol consumption of three drinks per day. Using the different methodologies and assumptions regarding alcohol contents employed in four highly respected studies, Turner found that the total alcohol amounts corresponding to three drinks per day ranged from 24 g to 48 g. Consequently, when reading an article that relates a certain number of drinks per day to a specific health benefit or risk, one must pay careful attention to how a drink is defined in that study. The wide methodological diversity helps to explain, at least in part, the seemingly contradictory study findings regarding the consequences of certain drinking levels. Miller and colleagues (1991) have extended Turner’s analyses by providing simple calculation rules for converting alcohol-consumption data among four standard drinking units currently used by researchers. The authors urge the adoption of a common method for reporting alcohol consumption.

## Assessing Alcohol Consumption

In addition to the problems associated with defining a drink, disputes over how alcohol consumption can best be assessed in population studies have hampered attempts to define moderate drinking. Some of those disputes stem from the fact that alcohol epidemiology incorporates characteristics of four major epidemiologic perspectives: (1) psychosocial epidemiology, (2) psychiatric epidemiology, (3) chronic disease epidemiology, and (4) epidemiologic sociology (Grant 1994). (See sidebar below for brief definitions of and differences among the four perspectives.)

**Epidemiology: Four Research Perspectives**

The body of research on alcohol epidemiology represents work from four major epidemiological perspectives: psychosocial epidemiology, psychiatric epidemiology, chronic disease epidemiology, and epidemiological sociology. Research from each perspective seeks slightly different information from study participants. The four disciplines thus complement each other in revealing drinking patterns and problems among the U.S. population.

***Psychosocial Epidemiology***

Psychosocial epidemiology and psychiatric epidemiology share common roots. Before World War II, both disciplines relied on key community informants, medical data experts, and agency records for information that defined alcohol-related trends in the population. Following World War II, a second generation of studies evolved that used written measurement instruments, psychiatrists’ evaluations of client profiles, and interviews.

The psychosocial epidemiology perspective holds that distinct psychiatric disorders, including alcohol-use disorders, are merely different manifestations of common etiological factors, particularly social stress. Psychosocial epidemiologists commonly rely on the psychometric tradition of psychology, wherein researchers depend on self-reports from subjects who answer multiple-choice questionnaires (Grant 1994).

***Psychiatric Epidemiology***

In contrast to psychosocial epidemiology, psychiatric epidemiology measures mental disorders, including alcohol-use disorders, primarily by categorizing them. By providing a category for alcohol-use disorders, this perspective accepts alcoholism as a medical disease. The psychiatric epidemiology perspective is based on psychiatry’s clinical interview tradition (i.e., interviews with patients). Most early clinical interviews either entirely excluded or poorly represented alcohol-use disorders, but current interviews do incorporate questions in those areas.^1^

***Chronic Disease Epidemiology***

Traditionally, chronic disease epidemiology has focused on such medical maladies as heart disease and cancer. Data on various chronic illnesses, as opposed to mental disorders, have been gathered since the turn of the century. Information on alcohol use, symptoms, and consequences, however, was not collected routinely until the early 1970s because alcohol dependence was not viewed as a chronic disease. By sponsoring regular surveys, the National Institute on Alcohol Abuse and Alcoholism (NIAAA) has played an important role in establishing alcohol dependence in this category.

***Epidemiological Sociology***

The perspective of epidemiological sociology is the synthesis of several epidemiological approaches to the study of alcohol use and abuse and their consequences. Here, use and consequences are studied independently rather than as one psychiatric condition. Systematic epidemiological sociological surveys of the general U.S. population began in the 1960s. Most of those national and community studies were sponsored by NIAAA and its predecessor within the National Institute of Mental Health. Since 1965 researchers at the Alcohol Research Group in Berkeley, California, have conducted, at approximately 5-year intervals, nine national surveys as well as numerous community studies. The researchers have invested much effort in maintaining some degree of comparability across surveys, despite changing definitions and conceptualizations of alcohol-use disorders (Grant 1994).

—Mary C. Dufour

1Changes over the past 25 years in the definitions of many psychiatric disorders have resulted in the continual need to develop new instruments to assess evolving criteria. For example, criteria for alcohol-use disorders appearing in the third edition of the *Diagnostic and Statistical Manual of Mental Disorders* (DSM–III), published in 1980, were modified significantly in the revised edition of DSM–III in 1987 and the DSM–IV in 1994 (American Psychiatric Association 1980, 1987, 1994). Likewise, criteria in the ninth revision of the *International Classification of Diseases* (World Health Organization [WHO] 1977) were modified substantially in the 10th revision (WHO 1992).

ReferencesAmerican Psychiatric AssociationDiagnostic and Statistical Manual of Mental DisordersThird EditionWashington, DCthe Association1980American Psychiatric AssociationDiagnostic and Statistical Manual of Mental DisordersThird Edition, RevisedWashington, DCthe Association1987American Psychiatric AssociationDiagnostic and Statistical Manual of Mental DisordersFourth EditionWashington, DCthe Association1994GrantBFEpidemiologyLangenbucherJWMcCradyBSFrankensteinWNathanPEAnnual Review of Addictions and Treatment3New YorkPergamon Press19947186World Health OrganizationInternational Classification of DiseasesNinth revisionGeneva, Switzerlandthe Organization1977World Health OrganizationInternational Classification of Diseases10th revisionGeneva, Switzerlandthe Organization1992

Research conducted using each perspective seeks slightly different information from study participants. Because different research traditions have different focuses, each tradition emphasizes different research questions, which may be hard to compare across studies. On the one hand, this diversity can be advantageous in that the four disciplines complement each other in revealing drinking patterns and problems. On the other hand, the variability also can be a handicap, because the information collected about alcohol consumption often is not comparable across studies. For example, one survey may ask questions in a way that permits a diagnosis of alcohol dependence. Another study, however, may ask questions about alcohol consumption and alcohol problems without including specific diagnostic criteria, and thus a diagnosis cannot be made.

A study’s proposed research goals dictate the particular measurement approach. Furthermore, techniques that are ideally suited for one population subgroup may not work equally well for other subgroups (e.g., teenagers versus senior citizens). Consequently, scientists conducting large and expensive population-based surveys to answer multiple research questions and elucidate drinking behavior in diverse population subgroups must weigh many factors in deciding which alcohol-consumption measures will best meet their needs. The accuracy and validity of the results regarding the quantity (i.e., number of drinks), frequency, and volume (i.e., drink size) of alcohol consumption depend primarily on two factors: the survey methodology and the assessment mode used.

### Survey Methodology

The effectiveness of an assessment instrument (i.e., survey questionnaire) in accurately determining drinking patterns is influenced by the way in which the survey questions are phrased, the order in which the questions are arranged, and the manner in which the answers are combined into a summary index (i.e., converted into a single measure for analysis purposes—for example, “moderate” drinker). The types of survey questionnaires most commonly used to measure alcohol consumption fall into five categories: (1) frequency measures, (2) quantity-frequency (QF) measures, (3) graduated frequency measures, (4) short-term recall methods, and (5) diary methods.

Another technique for assessing alcohol consumption is the timeline followback (TLFB) method (Sobell and Sobell 1995). The TLFB is a structured interview in which participants receive calendar-based memory cues to assist them in constructing a chronological report of their alcohol use. Although the procedure is widely employed in research on the efficacy of alcoholism treatment, the required interviews are highly individualized and, hence, generally impractical for use in large-scale population-based surveys.

Frequency measures query the respondent on his or her typical drinking frequency in a given timeframe (e.g., the past year), based on various predetermined categories from which to choose (e.g., “never,” “once a month,” “once a week,” or “everyday” ). Because these frequency measures do not assess the alcohol amount consumed on each drinking occasion, they do not allow researchers to calculate a person’s average or total volume of alcohol consumption.

QF measures query the respondent on both drinking frequency and average quantity consumed per occasion, thereby providing a measure of the total alcohol amount consumed. QF measures currently may be the most widely used instruments with which to measure drinking in most countries, including the United States. Generally, the quantity question asks for the typical number of drinks consumed per occasion, providing the respondent with some definition of a drink (e.g., one 12 oz can or bottle of beer) on which to base his or her answer. A popular variant of the QF methodology is represented by self-administered, semiquantitative food-frequency questionnaires, which assess the consumption of different foods (see, for example, the questionnaire developed by Willett and colleagues [1988]). For example, a simple frequency questionnaire would ask, “ How often do you drink milk?” A semiquantitative food frequency questionnaire, however, would ask, “ How often do you drink a glass of milk?” and may even define the size (e.g., 8 oz). In some QF surveys, respondents are asked how often and how much, on average, they consumed different types of alcoholic beverages over the past year. Those surveys generally include specific definitions of standard drink sizes for each beverage type assessed.

When analyzing the results of QF measures, researchers can use several formulas to multiply the frequency of alcohol consumption and the average amount consumed. One benefit of QF measures is that the analyses sometimes also provide information on drinking patterns. One disadvantage, however, is that respondents, particularly those with irregular drinking patterns, may have difficulty providing accurate answers, because they must mentally average their alcohol consumption over the entire year (Rehm 1998).

To overcome the problems associated with averaging alcohol consumption over an extended period of time, scientists have developed graduated frequency measures. Those questionnaires begin with a question eliciting the largest number of drinks consumed by the respondent on any one drinking occasion during the past year. Subsequent questions then ask about the number of occasions on which progressively lower alcohol quantities were consumed. This survey approach does not require as much mental calculation and recall by the respondent as do regular QF measures. The benefit of this approach is that reports of alcohol consumption are highly accurate. The main disadvantage, however, is that the greatly increased length of the questionnaire requires more time for respondents to answer and thus increases research costs.

Short-term recall methods ask respondents for information about their actual alcohol consumption over a short period of time (e.g., the past week). This approach is based on the assumption that respondents remember the actual amounts of alcohol that they consumed over short periods (e.g., the past week) more accurately than they remember the amounts consumed over long periods (e.g., 1 month or 1 year). The most commonly used measures in this category ask each participant to cite the number of drinks that he or she consumed on each of the 7 days preceding the survey, beginning with the most recent day (Rehm 1998). One drawback to this type of survey is that many infrequent or occasional drinkers may report no alcohol consumption during the time studied. Consequently, short-term recall measures may overestimate the proportion of abstainers compared with other survey methods.

To many people, the word “abstainer” means someone who drinks no alcohol. To others, including many researchers, the term may encompass more than nondrinkers, including some people who drink a little bit. Thus, the definition of abstainer may vary from study to study, and studies reporting higher numbers of abstainers often use a broader definition of “abstainer.” In the National Health and Nutrition Examination Survey I, to be classified as an abstainer, respondents had to have reported consuming less than one drink of beer, wine, or liquor in the previous year (Dufour et al. 1990). In contrast, in the National Longitudinal Alcohol Epidemiologic Survey, in order to be considered a current drinker, a person had to report consuming 12 or more drinks during the year preceding the survey interview. People consuming fewer than 12 drinks were classified as abstainers. Abstainers were further divided into former drinkers and lifetime abstainers. Former drinkers were persons who had consumed at least 12 drinks in a 12-month period sometime in their lives, but not during the 12 months immediately preceding the interview. Lifetime abstainers were those who had never consumed at least 12 drinks in a 1-year period (Dawson et al. 1995). Results from these two surveys may report different numbers of abstainers, not because of true differences in drinking practices but because of definitional differences.

In diary methods, participants record each drink consumed over a given timeframe (e.g., 1 week), ideally shortly after consumption. Researchers have recently introduced an automated variation of the diary method. In this approach, participants report their daily alcohol intake by calling a dedicated toll-free number and activating, through a touch-tone telephone, an automated, interactive voice-simulation system (Searles et al. 1995).

In summary, the five types of assessment instruments just described yield highly diverse data. For example, the assessed timeframe can range from the past 24 hours to the drinker’s lifetime. Similarly, the questions may assess general alcohol consumption or the individual consumption of specific beverage types (e.g., beer, wine, or spirits). The specific wording of questions also may vary among studies. Survey findings indicate that the more specific and detailed the questions are, the higher the reported consumption. Finally, the surveys may vary in scope:

Some surveys may address only alcohol consumption, whereas other surveys may assess all food and other nutrient intake, as well as additional health-related behaviors (e.g., smoking and exercise), and include only a few alcohol-specific questions.

Why are the differences among assessment instruments relevant to the discussion of moderate drinking? One reason is that for a given drinker, different questionnaires may elicit different responses and therefore lead to varying estimates of alcohol consumption for that person. Furthermore, even if a respondent provides identical answers, differences in the scientific assumptions and calculations associated with the survey methods may produce variations in the reported results. Studies comparing the results obtained with different assessment methods have noted many differences in findings, including the following (Rehm 1998).

Questionnaires using the graduated frequency approach consistently produce higher estimates of volume of alcohol consumption than do QF measures, particularly among heavier drinkers. One of the reasons underlying higher estimates with graduated frequency measures is that such measures generally involve more questions than do simple QF measures, particularly for heavier drinkers. Survey researchers have discovered that more questions (and consequently more answers) may lead to higher consumption estimates, which are generally considered to be more accurate.

Diary methods produce higher estimates than do either QF or short-term recall methods. For example, in the previously mentioned study using an automated interactive telephone reporting system (Searles et al. 1995), 50 volunteers reported their daily alcohol intake for 112 consecutive days. Other data collected by traditional means immediately after study completion demonstrated that drinkers—particularly heavier drinkers—retrospectively underreported their alcohol consumption.

More detailed and specific questions also elicit higher estimates of alcohol consumption. For example, separate QF questions for different periods within a given timeframe (e.g., each month within the past year) produce higher estimates than does one global QF question (e.g., consumption during the entire year). Similarly, beverage-specific questions or questions asking for consumption in different contexts (e.g., in bars, at home, or at parties and celebrations) produce higher estimates than do global questions asking about total alcohol consumption.

QF measures that assess not only typical alcohol amounts consumed per occasion but also the frequency and quantity of greater-than-normal alcohol consumption yield higher consumption estimates than do basic QF questions, particularly when the greatest amount consumed of each beverage is specified.

The reported alcohol consumption is likely to be higher if the respondent perceives the assessment to be less stigmatizing. For example, estimates of alcohol consumption are higher when alcohol-related questions are part of a food-frequency survey than when the same questions are posed in an alcohol-specific survey.

Familiarity with these methodological variations and their implications can help scientists and other interested readers understand and evaluate the wide discrepancies found across various studies that assess different drinking levels and their consequences.

### Assessment Mode

Alcohol surveys also vary in assessment mode—that is, in the way in which the survey is conducted (e.g., as a personal interview, self-administered questionnaire, or telephone interview) (Rehm 1998). In the past, most alcohol surveys were conducted via face-to-face interviews and therefore were labor intensive and expensive. The rapid progress in computer technology, however, has led to the development and use of computer-assisted telephone interview systems. Because they are considerably less costly than face-to-face interviews, telephone surveys are rapidly gaining popularity among survey researchers. Scientists are divided as to whether the assessment mode influences reported alcohol consumption. Recent studies have found no significant differences between in-person and telephone interviews on most measures of drinking behavior (Greenfield et al. 1997; Rehm 1998).

### Self-Reports and Their Limitations

Most survey methods used to calculate a person’s alcohol consumption are based on information reported by that person. Consequently, such surveys are subject to both intentional and unintentional errors of recall by the respondent, potentially resulting in inaccurate information. Although similar inaccuracies also can occur with self-reports of other health-related behaviors (e.g., the consumption of fruits and vegetables or the frequency of exercising), the impact of inaccurate reporting in studies of alcohol and other drug use may be particularly severe: One hallmark of alcohol dependence (and other addictions) is denial—that is, people who abuse or are dependent on alcohol often deny that they are having problems or that alcohol is at the root of those problems. Consequently, those people may grossly underestimate or lie about the quantity and frequency of their alcohol consumption.

For clinical purposes, however, accurate and reliable information about a person’s alcohol consumption is essential. For example, treatment providers base various treatment decisions on the drinking-behavior information provided by patients. Consequently, inaccurate information could result in suboptimal treatment. The relevance of accurate self-reports of alcohol consumption in general population studies, however, is a more complex issue. For many studies, researchers examining drinking practices in the general population may be satisfied with achieving ordinal validity—that is, with being able to consistently place people in the correct order of drinking levels (i.e., whether a person is at the lowest or highest end of the spectrum of alcohol consumption). For other purposes, such as establishing threshold levels or risk levels for alcohol-related health consequences, however, such an approach may not be sufficient. To establish the precise nature of the relationship between alcohol-consumption levels and the risk for developing a certain disease, it is crucial that researchers know the actual alcohol amounts consumed (Midanik 1982). Nevertheless, research to date investigating the association between alcohol consumption levels and various diseases has relied primarily on self-reports of alcohol consumption.

Despite the limitations of self-reports, studies examining the reliability and validity of survey measures of alcohol consumption have indicated high levels of reliability—that is, when asked more than once, people generally are consistent in how much alcohol they report using. In fact, in nutritional epidemiology studies that investigated the consumption of various food categories, reported alcohol intake was particularly reproducible compared with the reported intake of other nutrients (Longnecker et al. 1993). Validity estimates (i.e., estimates of whether the survey measures actually provide accurate information on drinking levels) are not always as high as reliability estimates, but they generally fall within the upper range of the validity estimates of many comparable research projects (Williams et al. 1985).

For some analyses, such as studies investigating drinking consequences (e.g., drinking and driving and other alcohol-related injuries and violence) not only the amount but also the pattern of alcohol consumption is important and should be assessed. For example, imagine two people who consume identical average volumes of alcohol (e.g., 14 drinks per week). One person consumes 2 drinks each evening, whereas the other person ingests all 14 drinks within a few hours on a Saturday night. That difference in drinking pattern has considerable implications for the drinkers with respect to the likelihood of experiencing negative outcomes, such as alcohol poisoning or alcohol-related traffic crashes. Unfortunately, little consensus exists among scientists as to what constitutes hazardous drinking and how one can best measure drinking patterns in general and hazardous drinking patterns in particular. Researchers have developed several definitions of hazardous drinking, such as consumption of five or more drinks on one drinking occasion or being intoxicated more than a certain number of times in a given time period. Few studies, however, have compared the ability of those various definitions to predict alcohol-related outcomes or their usefulness in shaping public health policy.

## What Is Moderate Drinking?

Not surprisingly, given the variability in the definitions of one drink, the numerous approaches to assessing alcohol consumption, and the subjective interpretation of the word “moderate,” definitions of “moderate drinking” vary considerably among researchers. In the English language, “moderate” can be used as both a qualitative and a quantitative term, but it generally carries strong qualitative connotations. For example, *Webster’s* dictionary (1966) defines moderate as “characterized by an avoidance of extremes of behavior; observing reasonable limits, showing discretion and self control” (p. 1451). Based on this definition, most people who consume alcohol would likely consider themselves moderate drinkers, regardless of the actual alcohol amounts they consume.

Despite the rather vague definition of “moderate,” alcohol survey researchers use the term to describe certain drinking levels. In their surveys, scientists must classify the wide range of alcohol consumption found in the population (e.g., from zero to more than 20 drinks per day) into a manageable number of drinking categories. One commonly used scheme includes the categories of abstainer, light drinker, moderate drinker, and heavy or heavier drinker. The definitions of each category, however, can vary among studies. For example, Dawson and colleagues (1995) proposed the following definitions, where one drink is equivalent to 0.5 fl oz alcohol:

- Abstainer: drinks less than 0.01 fl oz alcohol per day (i.e., fewer than 12 drinks in the past year)
- Light drinker: drinks 0.01 to 0.21 fl oz alcohol per day (i.e., 1 to 13 drinks per month)
- Moderate drinker: drinks 0.22 to 1.00 fl oz alcohol per day (i.e., 4 to 14 drinks per week)
- Heavier drinker: drinks more than 1.00 fl oz alcohol per day (i.e., more than 2 drinks per day).

To some degree, discrepancies in the definition of moderate drinking may result from the fact that some people confuse the term with “social drinking” — that is, drinking patterns that are accepted by the society in which they occur. Depending on the society, however, those drinking levels may not be moderate or risk free.

Even when a definition of moderate drinking has been developed, that definition may not apply equally to all people or under all circumstances. For example, although it may not be harmful for a party’s host to consume three or four drinks during the evening, the same amount of alcohol when consumed by a guest who plans on driving home could place the guest at risk for being in a car crash. Similarly, a healthy woman will likely experience no negative effects from drinking one drink per day; however, if the woman is pregnant, the same drinking level may lead to adverse effects (i.e., fetal impairment).

In addition to the circumstances under which drinking occurs, alcohol’s effects on the drinker (e.g., on the ability to drive a car) depend to a large extent on the blood alcohol levels (BALs) achieved after alcohol consumption. The same number of drinks, however, will result in different BALs in a 150 pound (lb) and a 250 lb person. Even people with identical body weights can achieve different BALs because of variations in the levels of water and fat in the body, which primarily depend on the drinker’s age and gender. Alcohol is a small, water-soluble molecule that is distributed throughout the body water. Women tend to have proportionately less body water and more body fat than do men and therefore may achieve higher BALs than do men with the same body weight after drinking the same alcohol amount. Similarly, body water generally decreases and body fat increases with increasing age. As a result of these physiological differences, the same number of drinks will result in different BALs in a 140 lb woman and a 140 lb man, or in a 20-year-old man and a 60-year-old man with identical body weights.

### Moderate Drinking Guidelines

One of the most compelling reasons for collecting data on alcohol consumption and for developing models of alcohol-related risks and benefits is the desire to determine “safe” or “ low-risk” levels and patterns of alcohol consumption^2^—that is, consumption levels below which drinking is not strongly associated with negative consequences. In fact, when people ask, “ What is moderate drinking?” what they often really want to know is how much alcohol is safe or sensible to drink or how much they can drink without being at high risk of incurring negative consequences.

Analyses of safe or low-risk drinking levels can help formulate public health policies, such as moderate drinking guidelines, which have been developed in many countries around the world. In the United States, such guidelines are included in the publication *Nutrition and Your Health: Dietary Guidelines for Americans*, a document produced jointly by DHHS and USDA. The *Dietary Guidelines*, which are updated every 5 years, are designed to inform the American public about food choices that promote health and prevent disease. With respect to alcohol consumption, the most recent edition states, “ If you drink alcoholic beverages, do so in moderation” (DHHS and USDA 1995). Moderation is defined as no more than one drink per day for women and no more than two drinks per day for men. A drink is considered to be 12 oz regular beer, 5 oz wine, or 1.5 oz 80-proof distilled spirits. Those drinking levels are considered a “ceiling,” not a “ floor” — that is, one can drink less than those levels and still consider oneself a moderate drinker.

The *Dietary Guidelines* also list several categories of people who should not drink at all. Those categories include children and adolescents, people who cannot keep their consumption moderate, women who are pregnant or trying to conceive, people who plan to drive or participate in activities that require attention or skill, and people using over-the-counter and prescription medications that interact with alcohol. Finally, the *Dietary Guidelines* provide specific recommendations for recovering alcoholics and for people who have family members with alcohol problems.

One of the challenges in developing effective moderate drinking guidelines is to communicate to the general population the plethora of underlying scientific data regarding alcohol’s impact on health. Several factors contribute to the difficulty of this challenge. First, many of the measures of alcohol use in scientific studies cannot be converted easily into information on what alcohol amount is “safe” to drink. Other studies do not consider consumption patterns. Second, many scientific studies are complex and are difficult to communicate effectively to the public. Third, numerous highly diverse risks and benefits are associated with different alcohol-consumption patterns, making it difficult to develop succinct yet precise guidelines. The more comprehensive the advice that is given, however, the harder it is to communicate that advice successfully. The average person does not read through pages of guidelines with numerous caveats and qualifications but prefers simple and unambiguous advice. Consequently, to enhance the likelihood that a wide audience will actually read and act according to certain guidelines, those guidelines must be short, concise, and reader friendly. For example, in the *Dietary Guidelines*, the alcohol guidelines take up only 2 pages in a 43-page, easy-to-read booklet.

Although concise guidelines are most likely to be effective, they are also associated with disadvantages. For example, such guidelines do not allow for delineating different definitions of moderate drinking to encompass individual variations or for describing the scientific underpinning on which the definitions are based. Instead, one definition of moderate drinking that applies to a broad segment of society must be used.

## Conclusions

This article has reviewed some of the difficulties associated with defining drinking levels both in general and for moderate drinking in particular. Some of the difficulties encountered stem from variations among researchers in how they define a standard drink and what drinking levels they consider to be “moderate.” Consequently, readers of scientific articles that explore various aspects of moderate drinking (including the remaining articles in this journal issue) must pay careful attention to the definitions used by each author.

Furthermore, although alcohol research in general is becoming increasingly sophisticated, the measurement of alcohol consumption remains imprecise. In alcohol epidemiology, differences abound in definitions, scientific assumptions (e.g., regarding the alcohol content of a drink), and methods for calculating drinking levels. Although no one method or assumption is inherently better or worse than another, substantial differences in their use and in the resulting findings exist. These differences may result in ambiguous or even conflicting results and must be taken into account in order to draw valid conclusions or develop appropriate guidelines.

Finally, many studies that examine alcohol consumption along with a multitude of other factors related to health outcomes are formulated and analyzed by chronic disease epidemiolo-gists and other researchers outside the fields of alcohol epidemiology and survey research. Those scientists may not be as attuned as alcohol researchers to the numerous methodological subtleties involved in measuring alcohol consumption and thus may be more likely to misinterpret some of the findings.

Although moderate alcohol consumption has long been sanctioned in American society, its objective risks and benefits are only now beginning to be quantified. The field of alcohol research, including studies on the effects of moderate drinking, is advancing at a dizzying pace. The development of methods for accurately determining people’s actual alcohol-consumption levels and drinking patterns will greatly enhance researchers’ ability to define moderate drinking and to elucidate the associated benefits and risks.

## Footnotes

1 One example of how the strength or alcohol content of a beverage can be standardized is the “ proof” measure, which generally is applied to distilled spirits. According to this measure, a 200-proof solution contains 100 percent alcohol by volume; an 80-proof beverage contains 40 percent alcohol by volume (Doernberg and Stinson 1985).

2 Because researchers do not know whether any type of alcohol consumption is actually “ safe,” some organizations (e.g., the Canadian Addiction Research Foundation and the Canadian Centre on Substance Abuse) have adopted the term “ low-risk drinking.” This terminology implies that certain drinking levels are not strongly associated with negative consequences and avoids use of the term “ safe drinking.”
